# Awareness, attitudes and perceptions regarding HIV and PMTCT amongst pregnant women in Guinea-Bissau– a qualitative study

**DOI:** 10.1186/s12905-017-0427-6

**Published:** 2017-09-04

**Authors:** Noel Vieira, Dlama Nggida Rasmussen, Inês Oliveira, Aureliano Gomes, Peter Aaby, Christian Wejse, Morten Sodemann, Lucy Reynolds, Holger W. Unger

**Affiliations:** 1 Association Ceu e Terras, Avenida do Brasil n. 7, Apartado 1257, 1031 Bissau Codex, Guinea-Bissau; 2 Bandim Health Project, INDEPTH Network, Apartado 861, 1004 Bissau Codex, Guinea-Bissau; 3 Department of Infectious Diseases, Odense University Hospital, Sdr. Boulevard 29, DK-5000 Odense, Denmark; 4 Statens Serum Institut, Artillerivej 5, DK-2300 Copenhagen, Denmark; 5 Department of Infectious Diseases, Aarhus University Hospital, Palle Juul-Jensens Boulevard 99, DK-8200 Aarhus, Denmark; 6 London School of Hygiene and Tropical Medicine, Keppel Street, London, WC1E 7HT UK; 7 Department of Obstetrics and Gynaecology, The Royal Infirmary of Edinburgh, Edinburgh, EH16 4SA UK; 8 Department of Medicine at the Doherty Institute, The University of Melbourne, Post Office Royal Melbourne Hospital, Parkville, Melbourne, VIC 3050 Australia

**Keywords:** HIV, PMTCT, Knowledge, Awareness, Barriers, Guinea-Bissau

## Abstract

**Background:**

The human immunodeficiency virus (HIV) continues to be a major cause of maternal and infant mortality and morbidity in sub-Saharan Africa. Prevention of mother-to-child transmission of HIV (PMTCT) strategies have proven effective in decreasing the number of children infected in utero, intrapartum and during the breastfeeding period. This qualitative study explores knowledge and perceptions of HIV amongst pregnant women, healthcare workers’ experiences of the national PMTCT services, and barriers to PMTCT, during a period of programme scale-up in urban Guinea-Bissau (2010–11).

**Methods:**

In-depth interviews were undertaken amongst 27 women and 19 key informants at local antenatal clinics and the national maternity ward in Bissau, Guinea-Bissau.

**Results:**

Amongst women who had been tested for HIV, awareness and knowledge of HIV and PMTCT remained low. Testing without informed consent was reported in some cases, in particular when the test was performed around the time of delivery. Possible drivers of inadequate counselling included lack of confidentiality, suboptimal healthcare worker training, lack of time, and perceived occupational risk. Demand-side barriers to PMTCT included lack of HIV and PMTCT knowledge, customary and cultural beliefs associated with HIV and ill-health, HIV stigma and discrimination, and fear of partnership dissolution.

**Conclusions:**

Socio-cultural and operational challenges, including HIV testing without informed consent, present significant barriers to the scale-up of PMTCT services in Bissau. Strengthening local capacity for effective counselling and testing in the antenatal setting is paramount. Further research into local customary beliefs relating to HIV is warranted.

**Electronic supplementary material:**

The online version of this article (10.1186/s12905-017-0427-6) contains supplementary material, which is available to authorized users.

## Background

The human immunodeficiency virus (HIV) is a principal contributor to the high burden of maternal and infant mortality and morbidity in sub-Saharan Africa [1–3]. In 2013 nearly 25 million people were living with HIV in the region (58% women) and there were an estimated 1.5 million new infections [4]; of these, 210,000 were due to mother-to-child transmission. Despite some progress, the incidence of paediatric infections remains high [4]. Functional prevention of mother-to-child transmission of HIV (PMTCT) programmes are key to reducing this number [4].

Several barriers to PMTCT have been observed in Sub-Saharan Africa at individual, community, national and international level [5–9]. These barriers commonly relate to the implementation and uptake of, and adherence to, PMTCT [10–12]. Individual, social and structural factors are all important determinants of PMTCT success, with stigma, difficulties with partner disclosure, perceived compulsory testing, confidentiality issues, inadequate counselling, challenges with healthcare worker attitudes and medical supply issues being major themes in recent studies [13–15].

Guinea-Bissau is a lusophone West African country with 1.7 million inhabitants, of which 300,000 reside in the capital, Bissau [16]. Its multi-ethnic population has endured frequent political instability. The country ranks amongst the poorest worldwide [16]. Maternal deaths (790 per 100,000 live births in 2010) and under-five mortality rates (129 per 1000 live births) are unacceptably high [17, 18]. Guinea-Bissau is affected by a generalised epidemic of HIV-1 and HIV-2 [19]. In a cross-sectional survey from 2006, 5% of adult residents of Bissau were seropositive [20] and 7% of pregnant women tested positive during the same period [21]. There were an estimated 6100 children (<15 years) living with HIV in Guinea-Bissau in 2014 [22]. In a survey from the same year, 80% of pregnant women attended antenatal clinical at least once and 70% of all pregnant women tested for HIV (reported combined HIV-1/2 prevalence 4.3%). Eighty two percent of seropositive women, and 33% of their children, ultimately received antiretroviral therapy [23].

At the time this study was conceived a national knowledge, attitudes, beliefs, practices (KABP) survey showed that 94% of pregnant women attended antenatal clinic at least once, yet only 4% had knowledge of vertical transmission (2008) [24]. PMTCT had been available at selected antenatal clinics (ANC) in Bissau since 2002 [21], but it was not until 2007 that PMTCT services started being introduced more widely across the capital. Simultaneously, opt-out HIV testing was offered at the national maternity ward in Bissau to women just before or after delivery as many had no prior antenatal care or HIV testing. A more recent KABP survey from 2014 indicates that awareness of vertical transmission amongst women has since improved (76%) [25].

There is a paucity of qualitative research regarding perceptions of HIV amongst pregnant women and barriers to the provision PMTCT in Guinea-Bissau. This study, which was conducted in 2010–11, aimed to evaluate perceptions and awareness of HIV and PMTCT amongst pregnant women, and to identify potential barriers to PMTCT in Guinea-Bissau.

## Methods

We conducted a qualitative, interview-based study using a preliminary focus group discussion (FGD), followed by in-depth interviews [26].

### Study site

The study was undertaken, between August 2010 and February 2011 in Bissau, at three public antenatal clinics (Bandim, Belem, Simao Mendes National Hospital [SMNH]) and the SMNH maternity ward. SMNH is the principal provider of comprehensive emergency obstetric care in Guinea-Bissau (~7000 deliveries annually). All of the country’s ethnic and religious groups are represented in Bissau. The Bandim Health Project (BHP), a health and demographic surveillance site and member of the International Network for the Demographic Evaluation of Populations and Their Health in Developing Countries (INDEPTH), served as a local platform for the research.

At the time this study was conducted, PMTCT was offered largely in accordance with the 2007 World Health Organisation guidelines [27]. This consisted of zidovudine (AZT) from week 28 of pregnancy, and single-dose nevirapine at delivery, and AZT plus lamivudine (3TC) at delivery and for a further 6 months postpartum, at which point women were advised to cease breastfeeding. Infants were provided with single-dose nevirapine and AZT for 7 days. Due to drug supply interruptions not all women and infants benefited from this regimen [28]. National policy has since moved towards Option B, i.e. the provision of antiretroviral therapy from the point of diagnosis of infection antenatally until cessation of breastfeeding, at which point women discontinue treatment (unless it is required for their own health). Infants receive treatment at delivery and prophylaxis throughout the duration of breastfeeding) [29].

In addition to an incremental PMTCT roll-out at ANCs in Bissau, counselling and testing was offered at the SMNH maternity ward, as many women presenting for delivery had not had prior antenatal care and/or HIV testing. Here, midwives performed frontline PMTCT duties. Women were counselled and tested before or after delivery, depending on the clinical circumstances. Women who tested positive were referred to a local non-governmental organisation (“Céu e Terras”), which provides comprehensive medical and psychosocial care and follow-up [21]. HIV-testing and antiretroviral treatment were free of charge. The principal investigator, through his involvement in setting up and maintaining a database of pregnant women undergoing HIV-testing at HNSM, saw a need to evaluate perceptions and awareness of HIV and PMTCT amongst pregnant women, and to identify potential barriers to PMTCT in Guinea-Bissau.

#### Focus group discussion

Recruitment of study participants for in-depth interviews was preceded by a FGD. Participants included a midwife, a national research assistant with experience in social research, a local obstetrician and two female elders. Findings from the FGD, together with information from other relevant qualitative research studies, were used to design a simple interview guide for participating women [5, 7, 8, 24, 30–40]. The FGD also enabled identification of a baseline set of key informants. Purposive and chain sampling was subsequently applied to recruit further key informants.

#### In-depth interviews

A total of 19 key informants were recruited to the study. These were as follows: formal healthcare providers involved in PMTCT (five midwives, six doctors, one social assistant); informal healthcare providers (three traditional health practitioners); two PMTCT programme managers; and two HIV-awareness officers. In addition, 27 women aged ≥18 years were recruited (8 at antenatal clinic and 19 postpartum at SMNH). Sample size was determined by budgetary and research time constraints. These constraints also led to a higher proportion of women being interviewed in the postnatal period (as opposed to antenatally), as this population was more accessible and more women could be recruited within the time frame of the study. Women were invited to join the study following group information sessions held at the recruitment locations. All participants were interviewed once. Women and key informants were provided with information regarding the broad research aim of the study (‘interview study to improve pregnancy and delivery care in Guinea-Bissau’) and the background and affiliation of the interviewers. Our piloted interview guide consisted of broad, open-ended questions (‘Please tell us about your visit to the antenatal clinic and what happened during the visit’; ‘Could you describe to us your delivery?’) (Additional file 1). This provided a simple framework to aid the initiation and flow of the interview and allow exploration of relevant themes during the in-depth interviews [41, 42].

Patient interviews were conducted in private locations by two local research assistants with social science research experience (Aureliano Gomes, Neusa Andersen) and fluent in Guinea-Bissau’s Kriol (a Portuguese-based Creole, the lingua franca). The principal investigator (Holger Unger) conducted key informant interviews with assistance from a local research assistant (Aureliano Gomes). Interviews lasted between 25 and 120 min and were recorded using a digital recording device. Verbatim transcriptions of interviews conducted in Kriol were subsequently translated into English by a research assistant and double-checked by a second research assistant. Socio-demographic and clinical characteristics were collected for participating women (*n* = 27), but not for key informants. Women’s HIV-status was recorded only when information was volunteered during interviews. Key informants were interviewed at their workplace.

#### Data analysis

The data were evaluated using the constant comparative method of content analysis [26, 43]. A basic coding network was created, extended and altered with the emergence of further themes as analysis progressed (data coded by two researchers). A final set of themes and sub-themes were created upon refinement and completion of analysis. Interview data was managed and analysed using NVivo (QSR International, Doncaster, Australia), and presented in accordance with COREQ guidelines for reporting qualitative research (Additional file 2) [44].

### Ethics

The Ethics Committee of the Ministry of Health of Guinea-Bissau (NCP6/2009) and the Ethics Committee of the London School of Hygiene and Tropical Medicine approved this study. Verbal informed consent was obtained from all participants.

## Results

### Background characteristics of participating women

Principal ethnic groups included Balanta, Pepel, Mandinga and Fula. The median age was 24 years (interquartile range 21–28, range 18–39). Nine women (33%) were nulliparous and most participants had some formal education (85%). Eight out of 19 women (42%) interviewed after delivery had attended antenatal clinic at least once. The majority (25/27, 93%) of women were registered as having been tested for HIV in the current pregnancy and prior to the interview (Table 1).

**Table 1:** Background characteristics of women participating in in-depth interviews (*n* = 27)

Interviewee	Religion	Education	Marital status	Co-wife	Gravidity	Parity	Counselled and tested
Antenatal clinic patients *(n = 8)*							
1	Muslim	None	Married	No	6	5	Yes
2	None	Primary	Separated	No	5	4	Yes
3	Christian	Secondary	Married	No	1	0	Yes
4	Animist	None	Married	No	2	1	No
5	Muslim	Tertiary	Single	-	1	0	No
6	Catholic	Tertiary	De facto	No	1	0	Yes
7^a^	Muslim	Secondary	Married	Yes	5	4	Yes
8	Muslim	Secondary	Married	No	3	0	Yes
Maternity patients *(n = 19)*							
9	Muslim	Primary	Married	No	2	1	Yes
10	Muslim	Primary	Married	No	1	0	Yes
11^a^	Animist	Secondary	Married	Yes	4	3	Yes
12	Muslim	Secondary	Single	-	1	0	Yes
13	Muslim	Secondary	Married	No	1	0	Yes
14	Christian	Secondary	De facto	No	2	1	Yes
15	Muslim	Primary	Married	No	4	3	Yes
16	Christian	Secondary	Single	-	1	0	Yes
17	Christian	Tertiary	Married	No	6	4	Yes
18	Muslim	Secondary	De facto	No	2	1	Yes
19	Animist	Secondary	Married	No	2	1	Yes
20	Christian	Secondary	Married	No	6	5	Yes
21^a^	Christian	Secondary	Married	No	5	4	Yes
22	Animist	Secondary	Single	-	1	0	Yes
23	Muslim	None	Married	No	2	1	Yes
24	Animist	Secondary	Married	No	4	3	Yes
25	Muslim	Primary	Married	No	2	1	Yes
26	Christian	Secondary	Married	No	3	2	Yes
27^a^	Animist	None	Married	No	4	3	Yes

*NA* not applicable. ^a^Women who voluntarily disclosed HIV-positive status during the interview

### Women’s knowledge of HIV/AIDS and PMTCT

Interviews suggested that HIV-related knowledge was incomplete, despite participants having undergone testing in the current pregnancy. While many women knew about the transmission of HIV through sexual intercourse (Table 2, Interviewee [Int]-2), knowledge regarding other means of transmission was less common. Some patients demonstrated knowledge of several routes of transmission, yet responses were often incomplete or erroneous. Mosquito bites, sharing plates, spoons and even clothes were reported as way of contracting HIV:

**Table 2:** Key quotes from patients and key informants

Category	Interviewee/key informant	Quote
Women’s knowledge of HIV/AIDS and PMTCT	Int-2	*I only know one way, it’s through sex. When someone is positive and you have sex with him, you can get it.*
	KI-1	*About PMTCT they [pregnant women] know almost nothing. A lot of explanations need to be given*. (midwife)
Attitudes to, and perceptions, of HIV	KI-3	*In Guinea-Bissau we view HIV as a bad thing…Those who have it will be very secretive, they would not tell anyone because of fear of discrimination.* (female HIV awareness worker)
	Int-27	*(Int-27) When I told my younger sister that I tested positive she and my uncle told me to change house and find my own place to stay. They said I had to leave because they worried they could also get infected.*
Experience of HIV counselling and testing for PMTCT	Int-14	*I went directly to the delivery room and delivered. Then they [healthcare workers] pricked my finger… They didn’t ask for permission and they didn’t say why they are doing this. It is better to tell what it [the test] is for but it’s not a problem that they didn’t tell me*
	Int-21	*They didn’t ask me to do the test…I think they did the test when I was asleep. They did it and then gave me the result, then they gave me some medicine*.
Difficulties with counselling, testing and treatment	KI-4	*We [midwives] try and talk to the patient with as few other people around as possible…We don’t have a counselling room, we talk to the patient when they come to deliver… I think counselling should take place at the antenatal clinic and when giving health advice…But most patients have not been to the antenatal clinic.* (midwife)
	KI-1	*Some of them [women] don’t know what it [HIV] means... sometimes there is not enough time to talk, when the woman is ready to deliver we must work quickly…it is quite difficult to convince someone to take the test…I need more training for counselling and to provide psychological support.* (midwife)
	KI-6	*When someone tests positive, and we are giving medicine to the mother and the child some people ask questions…they think something is wrong with that patient.*
		*I repair episiotomies and I haven’t had a needle stick yet, I always take good care...I am careful because I know I can get it [HIV] that way…One must protect oneself, especially when you have a positive woman, you put two pairs of gloves and you make sure you disinfect your hands afterwards…It would be great if we had more money for testing and gloves. Because we often don’t know whether the woman is positive…if she is contaminated we must take extra care.* (midwife)
Motivation for and against HIV testing	Int-8	*They refuse because they live another life outside of marriage. Some of them are not faithful. Since I married I have only been with my husband.*
	Int-17	*When you ask someone to do the test, and they refuse it is because they are afraid, afraid because she/he could have HIV because of their lifestyle*
	Int-25	*Those who decline the test are those who do not want to live a good life*
	Int-24	*Some women don’t want to test [for HIV] because of shame. If you are positive many people will say you are not faithful.*
	KI-9	*The majority of women agree to testing once they understand that this [HIV testing] is done so that their child does not get sick. The issue is with their partner...women are afraid of losing their marriage, being left without funds, and don’t know how to tell their husband. To be honest, even with counselling, we had some husbands filing for divorce.* (healthcare worker)
*Customary and cultural beliefs in relation to HIV/AIDS*	KI-10	*Some patients have the idea that AIDS is something that comes from traditions…. people try to find a justification for getting ill with AIDS, for example that it is because of a ceremony they haven’t done.* (healthcare worker)
	Int-27	*Many women say that when they are sick, their breast milk is not good. That’s why I believe my breast milk is not good for my baby.*

*AIDS* acquired immunodeficiency syndrome, *HIV* human immunodeficiency virus, *Int* interviewee. *KI* key informant, *PMTCT* prevention of mother-to-child transmission

*(Int-18) I heard you can get it through sex, sharp objects and even clothes. If you borrow clothes from someone who is positive, if she sweats a lot then you can get it.*

Only two of 27 women tested in the current pregnancy and prior to the interview mentioned vertical transmission. Midwives also observed that most women had very limited knowledge of PMTCT (Table 2; Key informant [KI]-1). One male healthcare worker explained:

*(KI-2) I think the reason for this is that they [women] have never seen it [manifest HIV], see to believe is what they say here. HIV is unlike malaria where you can see someone getting sick quickly…when a person is HIV positive you don’t see the illness for a while.*

Women who stated that they believed in the existence of HIV said they did so because they had seen or known someone suffering from HIV/AIDS, had learned about the infection and its sequelae during pre-test counselling, or had heard people in their communities talk about it. Although many women knew there was no cure, awareness and knowledge of disease-limiting treatment was sparse.

### Attitudes to, and perceptions of, HIV/AIDS

**Fig. 1:**
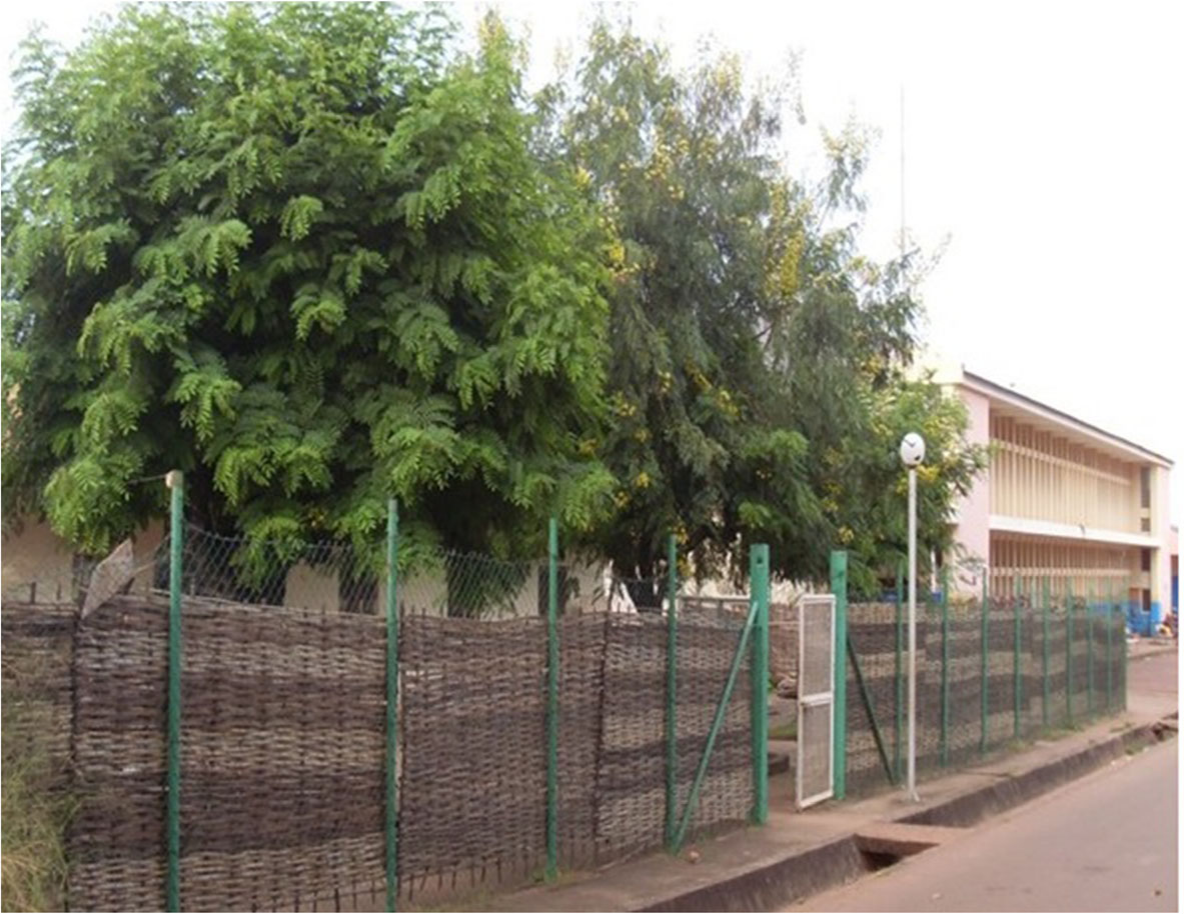
The former Bissau HIV clinic, located just adjacent to the Simao Mendes National Hospital Maternity ward, surrounded by a bamboo fence to provide ‘visual’ confidentiality

Interviews revealed that women frequently used the terms ‘*bad*’ or ‘*dangerous*’ when discussing HIV and People Living with HIV/AIDS (PLWHA) (Fig. 1). Women commonly reported on perceptions on PLWHA circulating in their local communities, rather than discussing their own. Here, being HIV positive was associated with unfaithfulness, immoral behaviour, or even criminal activity and prostitution (Table 2; KI-3). One maternity patient reported:

*(Int-13) People say that someone who has HIV is not a good person… that they do very bad things, like sleeping around… If you are positive many people will say that you are unfaithful…Someone who has the infection is often regarded a criminal or prostitute.*

**Fig. 2:**
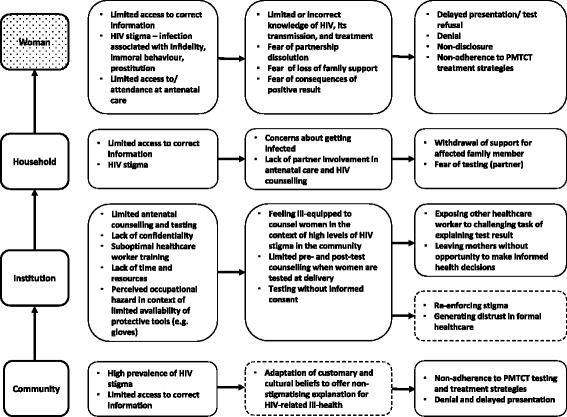
Findings for the in-depth interviews with women and key-informants. Dotted lines indicate themes observed in other, similar research

Two women participating in the interview study had been diagnosed with HIV before the current pregnancy. Both reported discrimination as well as abandonment by their former partners and/or family (Table 2; Int-27) (Fig. 2).

### Experience of HIV counselling and testing for PMTCT

Most women reported that they had finger prick sample taken around the time of delivery or during antenatal care, yet many did not know what this had been for. Some women reported having been tested (and treated) without consent, in particular when tested during labour or shortly after delivery (Table 2; Int-14, Int 21) (Fig. 2). A maternity patient recounted:

*(Int-12) Someone [at the hospital] did a finger prick but I wasn’t told what it was for…They didn’t say anything. They only said to hold up my finger… They had also asked me to do many tests at the antenatal clinic. I did them all but I don’t know if an HIV test was done.*

### Difficulties with counselling, testing and treatment

Key informants voiced concerns about peripartum HIV counselling and testing. Midwives felt that the lack of privacy, specifically the lack of a room to disclose test results, posed a significant challenge (Table 2; KI-4) (Fig. 2).

*(KI-5) We have one problem, there is no private room to do this [disclose a test result] so we have to wait until no one is around… I have to whisper to make sure that others don’t hear what I am saying.* (midwife).

Lack of time to perform counselling and testing, in particular when the woman was in advanced labour, was also a concern to staff, even though national guidelines recommend to defer counselling and testing in this circumstance (Table 2; KI-1). Focussing on antenatal counselling and testing was seen as a way to circumvent the lack of confidentiality in the delivery suite, and midwives recognised the general need to improve antenatal clinic attendance to make this happen (Table 2, KI-4). Midwives also highlighted their own difficulties with providing adequate counselling at delivery and felt that there was a need for more training (Table 2; KI-1). Confidential provision of antiretroviral treatment was also viewed as challenging (Table 2; KI-6).

Maternity workers were aware of occupational health risks including the risk of contracting HIV during clinical work, and expressed a desire to know a patient’s status in order to take extra precautions (Table 2; KI-6) (Fig. 2).

A healthcare worker involved with the follow-up of HIV-positive mothers described how patients recently diagnosed with HIV at SNMH often arrived with limited or no HIV-related knowledge, highlighting deficiencies in pre- and post-test counselling. Testing with limited or absent counselling caused affected women considerable distress, and posed a significant challenge to healthcare workers providing follow-up care and treatment for these patients:

*(KI-7) When I start talking to them [maternity patients] some start crying because they think they will die tomorrow...They don’t know much about HIV…They sometimes arrive having been given no information whatsoever…Even when they are doing the test antenatally, they don’t get much information. When they arrive here they often don’t know what they have come here for.* (healthcare worker).

This apparent lack of pre- and post-test counselling contradicted national guidelines in place at the time interviews were conducted, and indicates a significant health systems gap which prevents patients being able to access crucial information.

### Motivation for and against HIV testing

There were many reasons given for why women chose to be tested. Some viewed HIV-testing as an opportunity to find out about one’s health and stated that this motivated them to seek counselling and testing. While few of those interviewed were aware of vertical transmission, those who were, viewed protection of their child as a key motivator for testing. For some women testing was a means of highlighting fidelity and their character as a “good person” (Table 2; Int-8, Int-17, Int-25). Anticipating negative consequences was the most important reason for declining the HIV test and many of those interviewed particularly feared stigmatisation (Table 2, Int-24) (Fig. 2). In addition to stigmatisation, discrimination was perceived as a barrier to testing:

*(KI-8) When you find out you are positive and you live with another person they might discriminate against you and throw you out, this is why people don’t want to test. A personal example, when my family heard that I was positive they didn’t allow me to touch different items, even my daughter was not allowed to touch items in the house…They [women] feel bad, they have a lot of worries about this situation….* (female awareness worker).

Key informants thought that women who declined testing did so because it was the ‘safer’ option, even if this could mean missing out on treatment and preventing vertical transmission: fear of abandonment (partner, relatives) in the event of a positive test result was mentioned (Table 2; KI-9). Partner counselling and testing was not routinely offered at ANCs, and was impracticable at SMNH. Some women stated that they would need to consult their husband prior to agreeing to be tested. The decision to test did not always rest with the women, as a healthcare worker explained.

*(KI-2) Women decline HIV-testing because they are afraid of being abandoned by their partners… Sometimes the partner comes with her to the clinic. We ask about the test, he speaks and we don’t really know what she thinks. He then says that the test is not necessary*. (healthcare worker).

### Customary and cultural beliefs in relation to HIV/AIDS

Women and key informants mentioned traditional beliefs in relation to HIV/AIDS, with traditional beliefs and ceremonies occupying an important role in explaining ill-health (Table 2; KI-10). A maternity patient, who had tested positive at delivery, said:

*(Int-11) My husband has been sick for a long time. They [family and community members] said it is tarbessadu [traditional belief] that he has got.*

One key informant stated:

*(KI-11) Many people say HIV/AIDS does not exist and they call it bajudesa but it is the same as AIDS…The woman must do a ceremony before marrying. If she doesn’t she falls ill and dies.* (healthcare worker).

Tarbessadu described the event of a footling breech birth, and bajudesa meant that a woman had fallen pregnant or had a sexual relationship before traditional marriage. Both were associated with illness unless a traditional health practitioner had performed a ceremony. The illness associated with ‘untreated’ tarbessadu and bajudesa had features very similar to those women associated with AIDS, such as weight loss, hair loss, bone/joint pain and diarrhoea.

Healthcare workers also reported difficulties with supporting sero-positive mothers to breastfeed whilst receiving antiretroviral treatment:

*(KI-12) They don’t want to breastfeed until they have done a ceremony [with the traditional health practitioner]. Instead they choose to give contaminated milk [formula made with dirty water].* (healthcare worker).

When patients experienced illness, or when told they had an infection, they thought that giving breast milk would be detrimental to their baby (Table 2, Int-27) (Fig. 2).

## Discussion

Despite having been tested for HIV during pregnancy or shortly after delivery women had limited knowledge and awareness of HIV, and of PMTCT in particular. The study found several potential barriers to the provision of PMTCT including: HIV testing without adequate informed consent and counselling; gaps in HIV and MTCT knowledge among women; perceived stigma at a household and community level; and HIV-related cultural beliefs.

Previous qualitative research and KABP surveys conducted in Guinea-Bissau indicate significant gaps in HIV-related knowledge, and the present study confirms these findings. Low health literacy, i.e. difficulties with accessing and processing health-related information, was shown to impact on adherence to antiretroviral treatment amongst patients in Bissau [45, 46]. A more recent KABP survey, conducted three years after this study, indicated improvements in most knowledge indicators [25]. The present study was conducted at a time of a major roll-out of awareness activities and counselling and testing services in Guinea-Bissau, which may have contributed to the improvements noted in the aforementioned recent KABP survey, including a notable increase in the awareness of vertical transmission.

Only two of our study participants specifically mentioned vertical transmission, despite the fact that most women had undergone HIV-testing prior to their study interview. National policy demands pre- and post-test counselling, yet evidence from this study suggests that counselling was ineffective, inadequate, or even absent. A number of study participants remembered having had a test but were unclear as to its purpose. Although this may in part be explained by low health literacy, lack of effective counselling is a more likely explanation, thereby denying patients their chance to access and process crucial health information. This absence of effective counselling, especially at the maternity, made the job of the healthcare workers responsible for providing follow-up care and explaining the implications of a positive test result very challenging.

HIV-related stigma may have resulted in HIV-testing without informed consent and/or inadequate pre- and post-test counselling. Some maternity workers felt underprepared and described the counselling process as difficult. Midwives were also aware of the risk of needle-stick injuries and took extra precautions when assisting women known to be HIV-positive. Fear of infection, and HIV stigma and discrimination, may have been a another driver of suboptimal or absent counselling [47]. The adequacy and effectiveness of pre-and post-test counselling may also have been affected by the apparent lack of space to provide and discuss confidential information. Routine (opt-out) testing at the time of delivery had been introduced in Bissau in 2007 as many delivering women had no antenatal HIV counselling and testing. Opt-out testing is known to have advantages in the antenatal setting and provides an opportunity to educate and counsel women [48, 49]. However, adequate counselling and testing at time of delivery appeared to prove challenging as healthcare workers had the task of providing detailed information within a short time period to patients with limited knowledge of HIV and vertical transmission. Unless conditions for both patients and healthcare workers are improved, opt-out peripartum HIV counselling and testing should be abandoned or restricted to selected cases, and efforts to prevent vertical transmission should focus on improving antenatal counselling and testing instead. Testing without informed consent is unethical and drives HIV stigma. Together with the perceived lack of confidentiality at public healthcare institutions it also risks increasing negative attitudes towards health facility-based childbirth and care [50], and may thus increase maternal and infant mortality [51]. Routine opt-out testing at delivery in Bissau ceased towards the end of 2013, primarily due to a lack of funding.

Women stated that being seropositive was often considered a sign of sexual immorality, prostitution and even criminal activity in their communities. Such negative perceptions are significantly associated with discriminatory attitudes [52, 53]. Changing knowledge, attitudes and perceptions at a community level is imperative as this could reduce stigma and mitigate its negative impacts. HIV-related stigma emerged as a principal theme, with potentially deleterious effects for both patients and healthcare providers. At the patient level HIV-related stigma may adversely affect the uptake of testing as well as healthcare-seeking behaviour and test result disclosure, for fear of repercussions such as discrimination and partnership dissolution [54–56].

Traditional beliefs identified in our study provide a non-stigmatising explanation of HIV-driven ill-health and are likely to thrive in Guinea-Bissau, given the high burden of negative attitudes towards PLWHAs. Traditional treatments may be preferred or used in addition to conventional antiretroviral therapy as Western medicine is distrusted and does not deal with the social dimension of illness [57]. Traditional beliefs shift the responsibility for ill-health from the individual to superior forces, possibly allowing PLWHAs to remained integrated in their communities [57]. Beliefs such as *tarbessadu* may interfere with test- and treatment-seeking behaviour and treatment adherence in Bissau [46, 58]. Customary beliefs surrounding breastfeeding must be taken into account when counselling women about PMTCT policies including breastfeeding on antiretroviral therapy [59]. The need for further assessment of traditional belief systems and treatments in Guinea-Bissau was also recognised in the 2014 KABP survey which detected other traditional beliefs in relation to HIV and found that informal healthcare is frequently sought [25].

The study has several limitations. Firstly, the number of interviews was limited by budgetary and time constraints; it is plausible that some relevant themes remained undetected as a result. Furthermore, data collection was restricted to an urban setting, and not all of Guinea-Bissau’s ethnic groups were represented; these factors limit generalisation of the study findings. Secondly, we were unable to conduct a study amongst partners of pregnant women. Partner involvement was found important in similar settings, yet is commonly hindered by socio-economic and cultural factors [60–64]. Further research into attitudes towards, and strategies to improve, partner disclosure and testing in the context of PMTCT in Guinea-Bissau is needed: in 2014 partners of only 15% of women co-attended ANC, and only 5% of partners ultimately underwent HIV-testing [23]. Lastly, this research is based on interviews conducted in 2010–2011, and significant changes to PMTCT policy, including the roll-out of option B, have since taken place [23]. Nevertheless, the country continues to struggle to reach PMTCT testing and treatment targets [23]. HIV counselling and testing at SMNH during the peripartum period ceased in April 2013 due to funding constraints. National stocks-outs of HIV-tests have, since 2011, continued to hamper PMTCT efforts in Bissau (manuscript in preparation), and the roll-out of PMTCT services at antenatal clinics in Bissau and in other areas has remained suboptimal. Reports from the grey literature indicate a continuous need for qualitative research into barriers to PMTCT in Guinea-Bissau [65]. Despite its limitations the present study provides a first formal qualitative assessment of barriers to PMTCT in Guinea-Bissau.

## Conclusions

In the era of scale-up of PMTCT services and the introduction of more comprehensive PMTCT regimens (option B) significant socio-cultural and operational challenges need to be overcome, including HIV testing in the absence of informed consent. Strengthening local capacity for effective counselling and testing in the antenatal setting is paramount, as adequate counselling around the time of delivery is difficult to achieve. The delivery of PMTCT must be accompanied by strategies that improve general HIV and PMTCT knowledge, address stigma and discrimination and take into account local customary and cultural beliefs. Further research into their role, as well as the relevance and feasibility of partner counselling in improving PMTCT uptake and adherence in Guinea-Bissau, is needed.

## Additional file

Additional file 1: Interview guides (English, Portuguese, Kriol). (DOCX 33 kb)
Additional file 2: COREQ checklist. (DOCX 116 kb)

## Abbreviations

3TC: Lamivudine
AIDS: Acquired immune deficiency syndrome
ANC: Antenatal clinic
AZT: Zidovudine
FGD: Focus group discussion
HIV: Human immunodeficiency virus
Int: Interviewee
KABP: Knowledge, attitudes, beliefs, practices
KI: Key informant
PLWHA: People living with HIV/AIDS
PMTCT: Prevention of mother-to-child transmission of HIV
SMNH: Simao Mendes National Hospital, Bissau, Guinea-Bissau
