# Supplementary material for: Awareness, attitudes and perceptions regarding HIV and PMTCT amongst pregnant women in Guinea-Bissau– a qualitative study
Source: BMC Womens Health. 2017 Sep 4;17:71. doi: 10.1186/s12905-017-0427-6 (PMC5584044; doi:10.1186/s12905-017-0427-6)
Supplement: Additional file 1: — Interview guides (English, Portuguese, Kriol). (DOCX 33 kb) [file 12905_2017_427_MOESM1_ESM.docx]

Ficha 1 (kriol). Pacientes/Doentes

***Gravidez/Parto***

*Kontanu um bocado di es gravidez?*

*Bu tchiga di bai na consultas pre-natal (CPN) durante es gravidez?*

*Di fabur, falanu di bu CPN durante es gravidez I quê ku aconteci ki bias.*

*Na es tempo ku bu prenha, cantu bias ku bu visita CPN?*

*Pabia di quê ku bu bai/ Pabia di quê ku bu ca bai?*

*Quê ku bu pensa de CPN?*

*Bu ta bai djunto ku un alguim?*

*Kal qui assuntus ku ê papia ku bo e quim ku papia ku bo?*

*Ê fassiu alguns analises? Si ê fassiu el, bu sibi I cal coldadis, I pa quê?*

*Contam bu experiencias li na maternidadi.*

*Bu pudi falanu di bu parto, kuma ki kuri?*

***VIH/PTMF***

*Quê ku bu sibi sobre infeçons na gravidez?*

*Bu obi faladu di VIH ku SIDA? Quê ki significa pa bó?*

*Alguim tchiga di papia ku bo di VIH na es gravidez I antis di bu padi?*

*Quê ku bo combersa?*

*Kuma ku bu pudi panha VIH?*

*Cal qui maneras ku bu cunsi ku VIH ta camba di um alguim pa utro?*

*Kuma ku bu pudi livra di panha VIH?*

*Quê ku bu pensa di analise di VIH?*

*Bu lembra sib bu tchiga di fassi analise de VIH na maternidade?*

*Contanu pabia di ké qui ê fassiu analise di VIH.*

*Quê qui ê falau sobre analise?*

*Kuma ku bu ta sinti ku es analise?*

*Bu tchiga di papia di es assuntu ku pape di mininu antis?*

*Bu tchiga di papia di es assuntu ku pape di mininu dipus?*

*Bu tchiga di paga pa fassi es analise?*

*Na bu manera di pensa, quê pudi pui utrus mindjeris ta nega fassi analise di VIH?*

*Bu kunsi djintis ku tene VIH?*

*Bu pudi falanu di elis?*

VIH pudi tratado?

*Cal ki tratamento ku tem la?*

*Quim ku pudi fassi alguim es tratamentos?*

*Cantu ki ta custa?*

*Bu pensa kuma es tratamentos pudi fassi algum kussa?*

*I tem utros tratamentos ku ta fassidu, pa utrus guintis fora di hospital?*

*Si contra resultado di analise I positive:*

*Contanu quê ku passa na bu cabeça otcha ki bu toma bu resultados?*

*Bu conta alguim sobre es resultados?*

*Quim ku bu conta?*

*Kuma qui reagi?*

*Bu ta pensa bim tene mas utrus fidjus?*

*I tem algum kussa ku akontisi na es barriga ku partu qui pudi fassiu ntindi gravidez di utro manera?*

*Na ké ku ta fala di gravidez ku padi I ku analise di VIH, bu acha kuma I tem algum mindjoria na sistema di saude ku bu contenti ku el?*

*Bu tchiga di bai ialça mon/bota sorti na mon di djambakus?*

*Na cal coldadi situaçons ku bu pudi bai djambakus?*

*Quim ku ta bai djambakus?*

*I tem algum kussa na tradiçon ou na bu crenças ku tene ligason di VIH ku SIDA?*

***DEMOGRÁFICAS BÁSICAS***

*Cantu ano ku bu tene?*

*Abo I cal raça/entia?*

*Bu tene religion?*

*Kal qui bu religion?*

*Nunde ku bu mora nel gossi?*

*Bu ianda scola? Si contra I bardadi, até cal classe ku bu bai?*

*Cal qui bu tarbadjo? Bu ta ricibi na bu tarbadju?*

*Bu tchiga badja de prenha?*

*Cantu fidjus ku bu padi em total?*

*Cantu fidjus qui bu tene gossi?*

*Abo I casada?*

*Bu tchiga di casa badja ku un utro omi?*

*Bu tene cumbossas?*

*Bu tene omi/rapas na es momento?*

***FIM DI ENTREVISTA***

*Bu tene algum kussa qui bu misti ba puntanu?*

*I tem algum kussa ku bu ca ntindi ou ku bu ca tene certeza d’el?*

*I tem algum kussa ku ta dissau preokupada ou ku bu misti discuti ku nos ou ku um utro alguim na es estudo ou ku quilis ku ta cuida di bo? (scribe contactos qui sta na folheto di pacienti).*

*Discuti alguns faltas di conhecimento/concepçons errados ku notado durante entrevista. Si I précis, fassi mas aconselhamentos ku materiais de informaçon*

**PREGNANCY/DELIVERY**

Tell us a little bit about this pregnancy.

Have you been to an ANC in this pregnancy?

Please tell us about your visits to the antenatal clinic and what happened during them.

How many times have you visited the ANC in total in this pregnancy?

Why did you/did you not attend?

What do you think of the ANC?

Did someone accompany you?

What topics were discussed with you and by whom?

Were any tests done? If yes, do you know what they were for?

Tell me about your experience here at the maternity.

Could you describe to us your delivery?

**HIV/PMTCT**

What do you know about infections and pregnancy?

Have you heard of HIV and AIDS? What does it mean to you?

Did anyone discuss HIV with you in this pregnancy and before delivery? What was discussed?

How can you get HIV?

What do you know about the way in which HIV is transmitted between people?

How can you prevent getting HIV?

What do you think about testing for HIV?

Do you remember getting tested for HIV at the Maternity?

Tell us about the circumstances of getting the test.

What were you told about testing?

How did you feel about the test?

Did you discuss this with the baby’s father beforehand?

Did you discuss this with the baby’s father afterward?

Did you have to pay for the test?

Why do you think some women might not want an HIV test?

Do you know anyone with HIV? Can you tell us about them?

Can HIV be treated?

What treatments are there? Who can administer them? What do they involve? What do they cost? Do you think they work?

Are there any treatments provided by people other than doctors or nurses?

*If revealing test result as positive:*

Tell us what has been going through your mind since you got your results.

Did you tell anyone else of the results?

Who did you tell?

How did they react?

Are you planning to have more children in the future?

Is there anything that happened in this pregnancy and delivery that might make you approach future pregnancies differently?

With regards to pregnancy and birth and HIV testing, are there any improvements in the system that you would appreciate for the future?

Have you ever sought the advice of a Djambuco?

Under what circumstance would you go and consult a Djambuco?

Who would typically go and consult a Djambuco?

Are there any traditions or religious beliefs related to HIV and AIDS?

**BASIC DEMOGRAPHICS**

What is your age?

Which ethnic group do you belong to?

Are you religious?

What religious group do you belong to?

Where do you currently live?

Did you receive schooling and if so until what is the highest grade that you completed?

What work do you do? Is this a paid job?

Have you been pregnant before?

How many children have you delivered in total?

How many children do you have at the moment?

Are you married?

Have you been married in the past?

Are there cumbosas (co-wives) in your family?

Do you have a partner at the moment?

**END OF INTERVIEW**

Do you have any questions you would like to ask us?

Is there anything that has remained unclear or you are not sure about?

Is there anything you are worried about or you would like to discuss with us or with someone else involved in your care or in this study? (Point out contacts provided in Patient Information Leaflet)

*Discuss possible knowledge gaps/misconceptions that were observed during the interview. Provide further counseling and information material if required.*

Ficha 2 (português). Informantes**-**chave

*Se importa, de começar a explicar a sua função no trabalho?*

*Qual é o envolvimento do senhor com o programa da PTMF?*

*O senhor poderia explicar a atual política da PTMF na Guiné-Bissau?*

*O que é que o senhor sabe acerca dos esforços de PTMF na maternidade?*

*Ests envolvido/a no aconselhamento e/ou teste? Fale-nos acerca disso.*

*Fale-nos sobre a forma como abordas os seus pacientes para o teste de VIH.*

*O que diz aos paciente antes do teste?*

*Fale-nos das circunstâncias durante os quais o teste é feito.*

*Fale-nos de como lidas com o resultaso do teste.*

*O senhor envolve o parceiro no processo do teste?*

*Na sua experiência, já aconteceu que uma mulher recusa o teste de VIH / PTMF?*

*Se sim, o que o senhor acha que podem estar na origem disso?*

*Porque acha que algumas mulheres querem fazer o teste de HIV, especialmente durante a gravidez?*

*Existem algumas tradições ou crenças religiosas relacionadas com o VIH e SIDA?*

*Como é que o senhor vê o papel do djambakus?*

*Sob quais circunstâncias um pacientes procuraria um djambakus?*

*O que o senhor sabe acerca da legislação sobre o VIH?*

**Key informants**

Would you mind starting with explaining what your job role is?

What is your involvement with the PMTCT programme?

Could you explain the current PMTCT policy in Guinea-Bissau?

What do you know about PMTCT efforts at the Maternity?

Are you involved in patient counselling and/or testing? Tell me about this.

Tell us about how you go about testing your patients for HIV.

What do you tell the patient before the test?

Tell us about the circumstances during which the test is done.

Tell us about how you deal with the test result.

Do you involve the partner in the counselling process?

In your experience, do women refuse PMTCT HIV tests?

If so, what do you think are the reasons for this?

Why do you think some women want an HIV test done?

Are there any traditions or religious beliefs related to HIV and AIDS?

How do you view the role of Djambukus?

Under what circumstance would patients go and consult a Djambaku?

What do you know about legislation with regards to HIV?

*Ficha 3. Djambakus (Traditional Health Practitioners)*

*1. Bu pudi explicanu bu tarbadjo ku bu papel?*

*Pabia di quê ku pui djintis ta bim nunde bo?*

*Cal probulemas di saude ku ta tissi djintis nunde bo?*

*Quê qui bu ta fassi pa elis?*

*Bu ta trata probulemas relacionadus ku gravidez? Si I bardadi, cal di elis?*

*Kal ki bu pontu di vista sobre tarbadju di medicus ku infermeiras na hospital?*

*2. Di fabur,falanu sobre VIH/SIDA.*

*Bu tchiga di djuda alguim di VIH/SIDA? Kuma?*

*Kal qui causa di VIH?*

*I tem algum tratamento ou us di custumi ku sta relacionado ku VIH qui bu sibi d’el?*

*Quê qui bu sibi di VIH ku gravidez?*

**Traditional Health Practictioners (Djambucos)**

Would you mind starting by explaining your work and role?

What do people come to see you with?

What type of (medical) problems do people come to see you with?

What do you do for them?

Do you deal with pregnancy-related problems? If so, which?

What’s your view on the work of hospital nurses and doctors?

Please tell us about HIV /AIDS.

Have you helped someone with HIV/AIDS? How?

What is the cause of HIV?

Are there any treatments or practices related to HIV that you know of?

What do you know about HIV and pregnancy?
